# Prenatal diagnosis of a *de novo* pathogenic *HNRNPK* variant in a Chinese fetus with abnormal ultrasound soft markers: a case report

**DOI:** 10.3389/fgene.2025.1661743

**Published:** 2025-10-27

**Authors:** Yuying Zhu, Zhen Yang, Qiumin Zhu, Ke Wu, Junying He, Hongmei Zhou

**Affiliations:** ^1^ Quzhou Maternal and Child Health Hospital, Quzhou, Zhejiang, China; ^2^ Clinic Lab, BGI Genomics, Shanghai, China

**Keywords:** nuchal translucency, prenatal diagnosis, whole-exome sequencing, *de novo* variant, hnRNPK

## Abstract

**Background:**

Heterozygous pathogenic variants in *HNRNPK* cause Au-Kline syndrome (AUKS), a neurodevelopmental disorder characterized by congenital anomalies and developmental delay. Prenatal diagnosis of AUKS remains challenging due to nonspecific ultrasound findings, such as increased nuchal translucency (NT) and nuchal fold (NF), which overlap with other genetic conditions.

**Methods:**

Whole-exome sequencing (WES) was performed on a fetus exhibiting increased NT and NF thickening, alongside parental samples. Identified variants were validated by Sanger sequencing, with structural and functional impacts predicted using bioinformatic tools.

**Results:**

WES revealed a *de novo* heterozygous frameshift variant in *HNRNPK* (NM_031263.4: c.504_507del) in exon nine of 17, which has been deposited in the ClinVar database with accession number VCV003899365 and classified as pathogenic (P). This variant results in a truncated protein (p.Lys168AsnfsTer35): bioinformatic predictions indicate the resulting mRNA is likely subject to nonsense-mediated mRNA decay (NMD), and any escaping mRNA would produce a severely truncated protein lacking critical functional domains, rendering it nonfunctional. Sanger sequencing confirmed the variant was absent in both parental genomes. Ultrasound findings aligned with AUKS-associated nonspecific prenatal anomalies, and post-induction gross examination confirmed subtle AUKS-related craniofacial features, expanding the known prenatal phenotype of AUKS and providing phenotypic severity context.

**Conclusion:**

Structural and functional analyses provide mechanistic insights into the variant’s pathogenicity, highlighting *HNRNPK*’s role in fetal development. These findings advocate for integrating genomic and phenotypic data to improve prenatal diagnosis of rare genetic syndromes.

## Introduction

Prenatal detection of abnormal NT and NF thickening are critical soft markers prompting investigation for underlying genetic disorders (Gadsboll et al., 2025; Sun et al., 2023). While commonly associated with aneuploidies, these findings increasingly signal the need to explore monogenic syndromes, particularly when standard cytogenetic and microarray analyses are normal.

Neurodevelopmental disorders (NDDs) represent a heterogeneous group of conditions with complex genetic etiologies. Recent advances in genomic technologies have enabled the identification of numerous causative genes, yet diagnostic challenges persist due to phenotypic variability, overlapping clinical features, and the high prevalence of variants of uncertain significance (VUS). Among these, disorders linked to RNA-binding proteins (RBPs) have emerged as critical players in neurodevelopment, given their central roles in RNA processing, chromatin remodeling, and gene expression regulation (Yamamoto, 2021). Among these, disorders linked to RNA-binding proteins (RBPs) have emerged as critical players in neurodevelopment, given their central roles in RNA processing, chromatin remodeling, and gene expression regulation (Parra and Johnston, 2022). Heterogeneous nuclear ribonucleoprotein K (HNRNPK), a member of the RBP family, is essential for multiple cellular processes, including transcriptional regulation and post-translational modifications (Xu et al., 2019). Pathogenic variants in *HNRNPK* are associated with AUKS (OMIM #16580), a rare multisystem disorder characterized by developmental delay, congenital anomalies, and distinctive craniofacial features (Choufani et al., 2022).

Prenatal ultrasonography plays a pivotal role in early detection of fetal anomalies, with soft markers such as increased NT and nuchal fold thickening serving as critical red flags for underlying genetic disorders. While these markers are classically associated with aneuploidies, their presence increasingly prompts investigation for monogenic syndromes, particularly when combined with normal karyotyping and microarray results (Wang C. et al., 2022). The biological basis of abnormal nuchal fluid accumulation often relates to altered extracellular matrix composition, cardiovascular dysfunction, or lymphatic dysplasia-processes implicated in multiple neurodevelopmental disorders.

AUKS was first described in 2015 in individuals with heterozygous loss-of-function (LoF) variants in *HNRNPK* (Au et al., 2015). Since then, the phenotypic spectrum has expanded to include variable presentations, such as cardiac, genitourinary, and skeletal abnormalities, often overlapping with other syndromes like Kabuki syndrome (Au et al., 2018; Workalemahu et al., 2023; Zhang et al., 2024). Notably, AUKS phenotypic severity correlates with variant type: postnatal studies show frameshift/nonsense variants are associated with more severe neurodevelopmental delay and craniofacial anomalies, while missense variants often present with milder cognitive impairment and fewer structural defects (Gillentine et al., 2021; Miyake et al., 2017). Despite growing recognition of AUKS, prenatal diagnosis remains challenging due to nonspecific sonographic findings, such as increased NT or nuchal fold thickening, which are also associated with other genetic and structural anomalies. Notably, the contribution of these ultrasound markers to the prenatal suspicion of AUKS has not been systematically characterized, and their association with *HNRNPK* dysfunction remains unexplored. To date, most reported *HNRNPK* variants are *de novo*, including missense, frameshift, and intronic variants, complicating variant interpretation without robust functional validation (Gillentine et al., 2021; Miyake et al., 2017).

Here, we report the prenatal identification of a *de novo HNRNPK* variant in a fetus with increased NT and nuchal fold thickening, expanding the phenotypic spectrum of AUKS to include early sonographic markers. This case underscores the importance of integrating prenatal imaging with exome sequencing and functional assays to improve diagnostic accuracy for rare genetic syndromes, particularly for nonspecific soft markers where standard testing yields negative results. Our findings also contribute to the growing evidence of *HNRNPK*-related disorders and highlight the need for further research into epigenetic signatures to facilitate prenatal and postnatal diagnosis.

## Materials and methods

### Ethical approval and sample collection

The study protocol received ethical approval from the Institutional Review Board of Quzhou Maternal and Child Healthcare Hospital. Written informed consent was obtained from both parents prior to sample collection. Prenatal amniotic fluid samples (15–20 mL) were collected under ultrasound guidance at 18+3 weeks, while peripheral blood samples (5 mL) were drawn from each parent for comparative genomic analysis. Genomic DNA was extracted from all samples using a commercial DNA isolation kit according to the manufacturer’s protocol (QIAamp DNA Blood Mini Kit, Qiagen). DNA concentration was 50–100 ng/μL with A260/A280 ratio 1.8–2.0.

### Clinical preliminary tests

To evaluate fetal genetic health, invasive prenatal testing was prioritized given the pregnant woman’s history of prior fetal demise (detailed in Case presentation). At 18+1 week of gestation, conventional G-banded karyotype analysis was performed on amniotic fluid samples collected under ultrasound guidance to exclude chromosomal aneuploidies and structural abnormalities. The analysis followed standard cytogenetic protocols: metaphase chromosomes were prepared from amniotic fluid cells, stained with Giemsa, and visualized under a microscope with a resolution of 400–550 bands (Xiao et al., 2016).

### WES and data processing

WES was performed on the BGI-seq2000 platform following exome capture using the Agilent SureSelect Human All Exon V6 kit. The sequencing achieved an average depth of 263× with 98.75% of target regions covered at >20× depth. Raw sequencing data underwent stringent quality control including adapter trimming using Cutadapt (v2.10) (Martin, 2011) and quality filtering with FastQC (Brown et al., 2017). Clean reads were aligned to the GRCh37/hg19 human reference genome using BWA-MEM (v0.7.17) (Li H. and Durbin, 2009), followed by duplicate marking and local realignment using GATK (v4.1.9.0) (Van der Auwera et al., 2013). Whole-exome sequencing of the proband achieved 99.89% coverage of coding regions, encompassing 58,682,415 bp across 25,701 genes. The mean sequencing depth reached 263.37x, with 98.75% of target regions exceeding 20x coverage.

### Variant calling and quality control

Variant calling was performed following GATK best practices (v4.1.9.0) (Van der Auwera et al., 2013), including HaplotypeCaller for initial variant detection and Variant Quality Score Recalibration (VQSR) for quality filtering (with training sets of HapMap 3.3 and 1000 Genomes phase 3 (Genomes Project et al., 2015). Single nucleotide variants (SNVs) and small insertions/deletions (indels) were jointly called across all samples. Variant annotation was performed using ANNOVAR (v20200608) (Wang K. et al., 2010), incorporating information from the following databases: gnomAD (v3.1.2) (Karczewski et al., 2020); 1000 Genomes Project (phase 3) (Genomes Project et al., 2015); dbSNP (v155) (Sherry et al., 2001). All databases were accessed on 2024–03–15. Variant quality was further assessed using quality metrics such as QUAL score (>30), QD (>2), FS (<60), and ReadPosRankSum (>−8.0).

### Variant filtering and prioritization

A multi-step variant filtering strategy was implemented to identify potentially pathogenic variants. Common variants (minor allele frequency, MAF >0.1%) were excluded based on gnomAD (v3.1.2) (Karczewski et al., 2020) and 1000 Genomes Project (phase 3) (Genomes Project et al., 2015) population frequencies. Remaining variants were prioritized based on predicted functional impact, including: 1. Protein-truncating variants (nonsense, frameshift, canonical splice site variants, defined as ±1/2 bp of exon-intron boundaries); 2. Missense variants with CADD score >20 (Rentzsch et al., 2019); 3. Variants in genes associated with relevant phenotypes in OMIM (v2024-03) (Amberger et al., 2019) or ClinVar (v2024-03) (Landrum et al., 2018). Inheritance patterns were carefully evaluated using pedigree information, with particular attention to *de novo* and compound heterozygous variants.

### Sanger sequencing validation

Candidate variants were validated using sanger sequencing. PCR primers were designed using Primer3 (v2.4.3) (Untergasser et al., 2012) to amplify 150–300 bp fragments encompassing each variant. Amplification was performed using Taq DNA polymerase under optimized conditions: 95 C for 5 min; 35 cycles of 94 C for 30 s, 58 C for 30 s, 72 C for 30 s; final extension at 72 C for 5 min. PCR products were purified using the QIAquick PCR Purification Kit (Qiagen, Hilden, Germany) and sequenced bidirectionally on an ABI 3730xl Genetic Analyzer (Thermo Fisher Scientific, Waltham, MA, USA) with the BigDye Terminator v3.1 Cycle Sequencing Kit. Sequence traces were analyzed using CodonCode Aligner (v10.1.1) (CodonCode Corporation, Centerville, MA, USA) to confirm variant presence and zygosity.

### Bioinformatic and functional analysis

Structural modeling was conducted using Phyre2 (v2.0) (Kelley et al., 2015) with the “intensive mode” to predict potential impacts of the variant on protein structure and function. Variant pathogenicity was classified according to the ACMG/AMP guidelines, incorporating evidence from population frequency, computational predictions, SIFT (Ng and Henikoff, 2003), functional domains, and phenotypic correlation.

### Statistical analysis and variant interpretation

All statistical analyses were conducted using R (v4.0.3; Available from: https://www.R-project.org/), with ggplot2 (Maag, 2018) for visualization and vcfR (v1.12.0) (Knaus and Grunwald, 2017) for VCF file processing. Variant classification was based on five lines of evidence: 1) population frequency data; 2) computational prediction scores; 3) inheritance pattern; 4) functional domain overlap (Paysan-Lafosse et al., 2025); 5) phenotypic correlation with known diseases (OMIM v2024-03) (Amberger et al., 2019). Final variant interpretation was performed by a multidisciplinary team to comprehensively evaluate potential genetic contributors to the observed phenotype.

### Protein structural analysis

Protein structural analysis of HNRNPK was conducted using the STRING database (Available from: https://cn.string-db.org/). This platform predicted three-dimensional interaction networks by integrating experimentally validated physical associations, pathway co-occurrence data, and computational binding models, identifying topologically critical binding partners and functional modules relevant to HNRNPK’s KH domain-mediated RNA recognition.

## Result

### Case presentation

**FIGURE 1:**
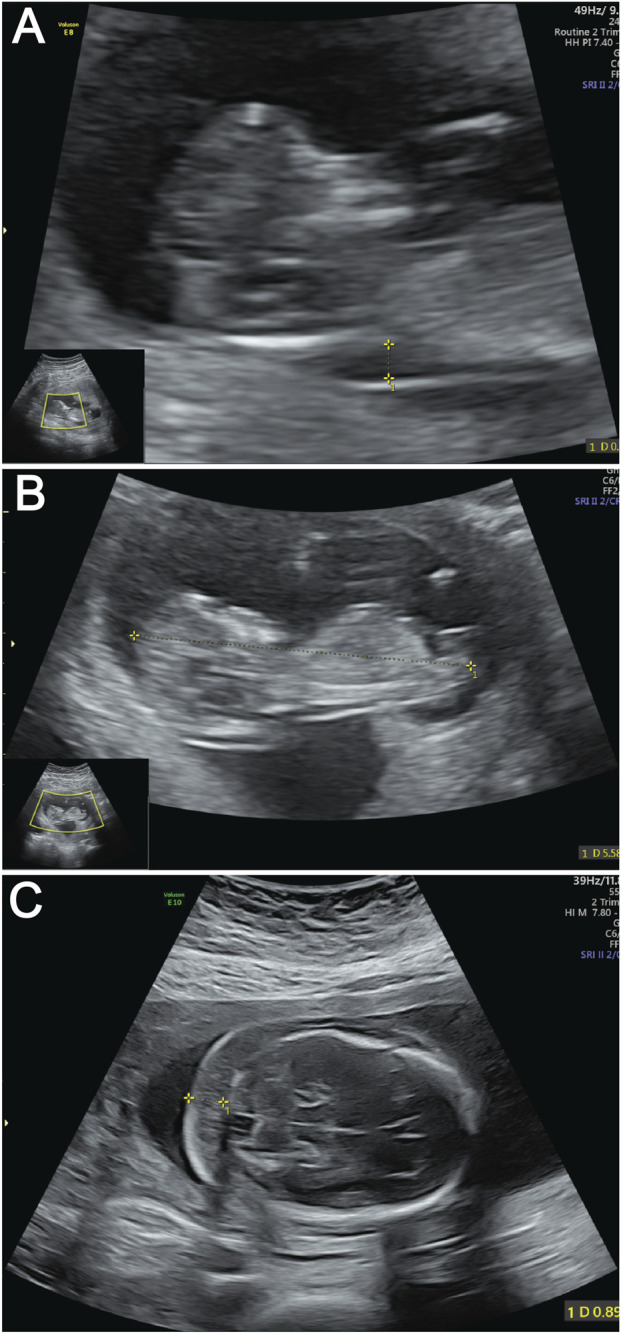
Ultrasound images of fetus demonstrating key anatomical views. **(A)** Ultrasound measurement of NT thickness. Yellow calipers mark the NT measurement points; **(B)** Fetal CRL measurement by ultrasound. The dotted line between yellow calipers indicates the CRL. **(C)** Ultrasound evaluation of NF thickness. Yellow calipers demarcate the NF measurement area.

A 30-year-old primigravida (gravida 1 para 0) with regular 30-day menstrual cycles (last menstrual period: 25 October 2024) and a history of one prior fetal demise has been confirmed pregnant following amenorrhea. During first-trimester screening, a significantly increased nuchal translucency (NT) measurement of 3.4 mm was detected (Figure 1A). Fetal biometry demonstrated appropriate growth parameters for gestational age: biparietal diameter 47 mm, head circumference 188 mm, abdominal circumference 172 mm, femur length 34 mm, and humerus length 32 mm (Figure 1B).

At the current presentation, a detailed fetal anatomical survey performed at this presentation confirmed the presence of a thickened nuchal fold measuring 9 mm (Figure 1C). Additional findings included a hyperechoic intracardiac focus (EIF) in the left ventricle, a hyperechoic uterine band bridging the anterior and posterior uterine walls (noted incidentally), and a transverse cerebellar diameter and lateral ventricular width within normal limits. Comprehensive structural evaluation confirmed normal anatomy of the fetal skull, spine, lips, extremities, stomach, kidneys, bladder, and umbilical cord insertion site. Cardiac anatomy appeared balanced with a normal four-chamber view and comparable great vessel dimensions. Fetal cardiac activity was regular at 129 beats per minute, with normal fetal movements observed. The patient’s main concern was the prior fetal demise and new ultrasound findings, which prompted referral to our fetal medicine clinic for further evaluation.

The placenta was anteriorly located, with a thickness of 21 mm and Grade I maturity. Amniotic fluid volume was normal. The Amniotic Fluid Index (AFI) was calculated as follows: Right Upper Quadrant 61 mm, Left Upper Quadrant 36 mm, Right Lower Quadrant 57 mm, and Left Lower Quadrant 38 mm. Umbilical artery Doppler velocimetry showed indices within the normal range, including a Pulsatility Index of 1.29, a Resistance Index of 0.71, and a Systolic/Diastolic ratio of 3.47. Post-induction examination revealed subtle craniofacial features, such as a broad nasal bridge and micrognathia, which are consistent with postnatal AUKS phenotypes. These postnatal features are in line with the phenotypic descriptions of AUKS in previously reported cases in the literature (Au et al., 2018; Workalemahu et al., 2023; Zhang et al., 2024).

### Identification of a *de novo HNRNPK* variant in the fetus

**FIGURE 2:**
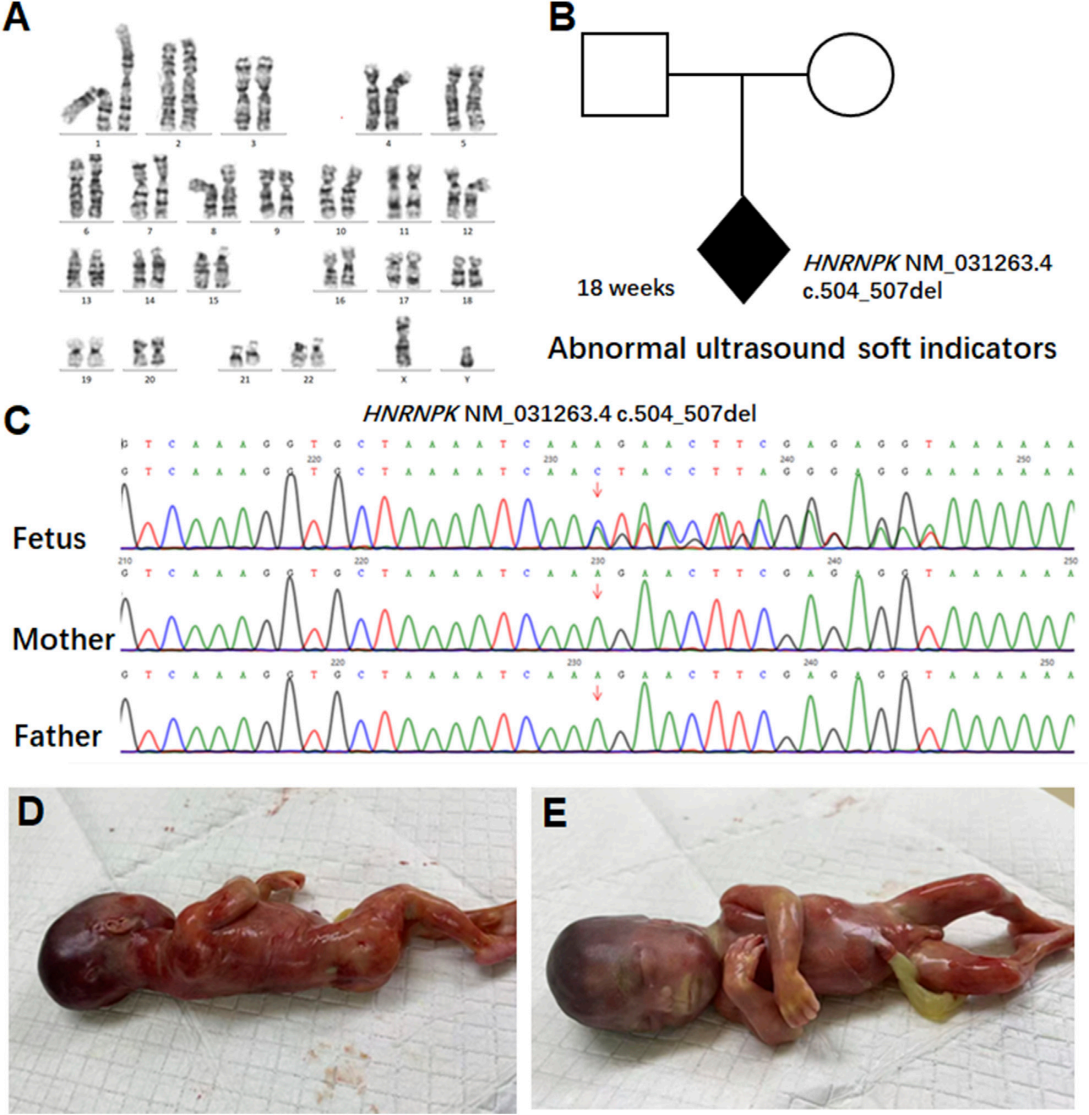
Pedigree analysis and genetic findings of the *HNRNPK* variant. **(A)** G-banded karyotype analysis of amniotic fluid cells from the 18-week fetus. Conventional G-banded karyotype analysis of amniotic fluid cells revealed a normal 46, XY karyotype. **(B)** Pedigree analysis of a family carrying an *HNRNPK* variant. The proband (shaded diamond, 18-week fetus) carries a heterozygous *HNRNPK* deletion (NM_031263.4:c.504_507del) in exon nine of 17. Parental symbols show no variant carriage. **(C)** Sanger sequencing validation of the *HNRNPK* variant. Chromatograms compare the proband (fetus) with parents at the variant site (arrow). The heterozygous 4-bp deletion appears *de novo* in the fetus, as both parents show wild-type sequences at this locus. **(D–E)** show the appearance of the 18-week-old fetus.

Given the history of prior fetal demise, serum quadruple screening was not performed; instead, invasive prenatal testing via amniotic fluid sampling was conducted at 18+1 week of gestation. Conventional G-banded karyotype analysis of the amniotic fluid sample revealed a normal 46, XY karyotype, ruling out common fetal chromosomal aneuploidies and major structural chromosomal abnormalities (Figure 2A).

To explore the genetic etiology underlying the fetus’s abnormal ultrasound soft markers, we performed WES on the fetal amniotic fluid sample and peripheral blood samples of both parents. WES results identified a *de novo* heterozygous frameshift variant in the *HNRNPK* gene (NM_031263.4: c.504_507del). This variant has been deposited in the ClinVar database (accession number: VCV003899365) and is annotated as pathogenic (P) therein, though no prior clinical case reports or literature citations are associated with it in the database. This variant is localized to exon nine of the 17 exons in *HNRNPK*-a region with critical functional significance, as exon nine encodes the SMART KH domain (Figure 2B).

To explore the genetic etiology underlying the fetus’s abnormal ultrasound soft markers, we performed WES on the fetal amniotic fluid sample and peripheral blood samples of both parents. WES results identified a *de novo* heterozygous frameshift variant in the *HNRNPK* gene (NM_031263.4: c.504_507del). This variant has been deposited in the ClinVar database (accession number: VCV003899365) and is annotated as pathogenic (P) therein, though no prior clinical case reports or literature citations are associated with it in the database.

At the molecular level, the c.504_507del variant induces a frameshift by deleting 4 consecutive base pairs in the coding sequence of exon 9. This deletion disrupts the normal reading frame of the *HNRNPK* gene, starting from the lysine residue at amino acid position 168 (Lys168) of the wild-type protein, the frameshift introduces a series of non-native, abnormal amino acids into the polypeptide chain. Subsequent translation is prematurely terminated at amino acid position 202, where a premature stop codon (PTC) is generated.

Sanger sequencing was performed to validate the variant, and chromatograms confirmed that the c.504_507del variant was present exclusively in the fetal sample (Figure 2C) and absent in both parental genomes-confirming its *de novo* origin. Given the pathogenicity of this *HNRNPK variant* consistent with AUKS etiology, and the persistent abnormal ultrasound soft markers, the pregnancy was terminated at 21 weeks of gestation. Post-induction gross examination of the fetus (Figures 2D,E) revealed phenotypic features consistent with AUKS, including a broad nasal bridge and micrognathia, which further supported the pathogenicity of the identified *de novo* variant. Notably, this fetus lacked major structural anomalies typical of severe postnatal AUKS, representing a mild prenatal phenotypic presentation despite the severe nonsense variant, consistent with age-dependent expressivity of AUKS (Kingdom and Wright, 2022). At 4-week post-termination follow-up, parents reported reduced anxiety and no depression symptoms; they expressed satisfaction with the diagnostic timeline. In reviewing the diagnostic and clinical management pathway of this case-from initial prenatal screening to post-termination follow-up-key milestones that guided decision-making are systematically summarized in Supplementary Table S1.

### Frameshift variant causes severe structural and functional defects

**FIGURE 3:**
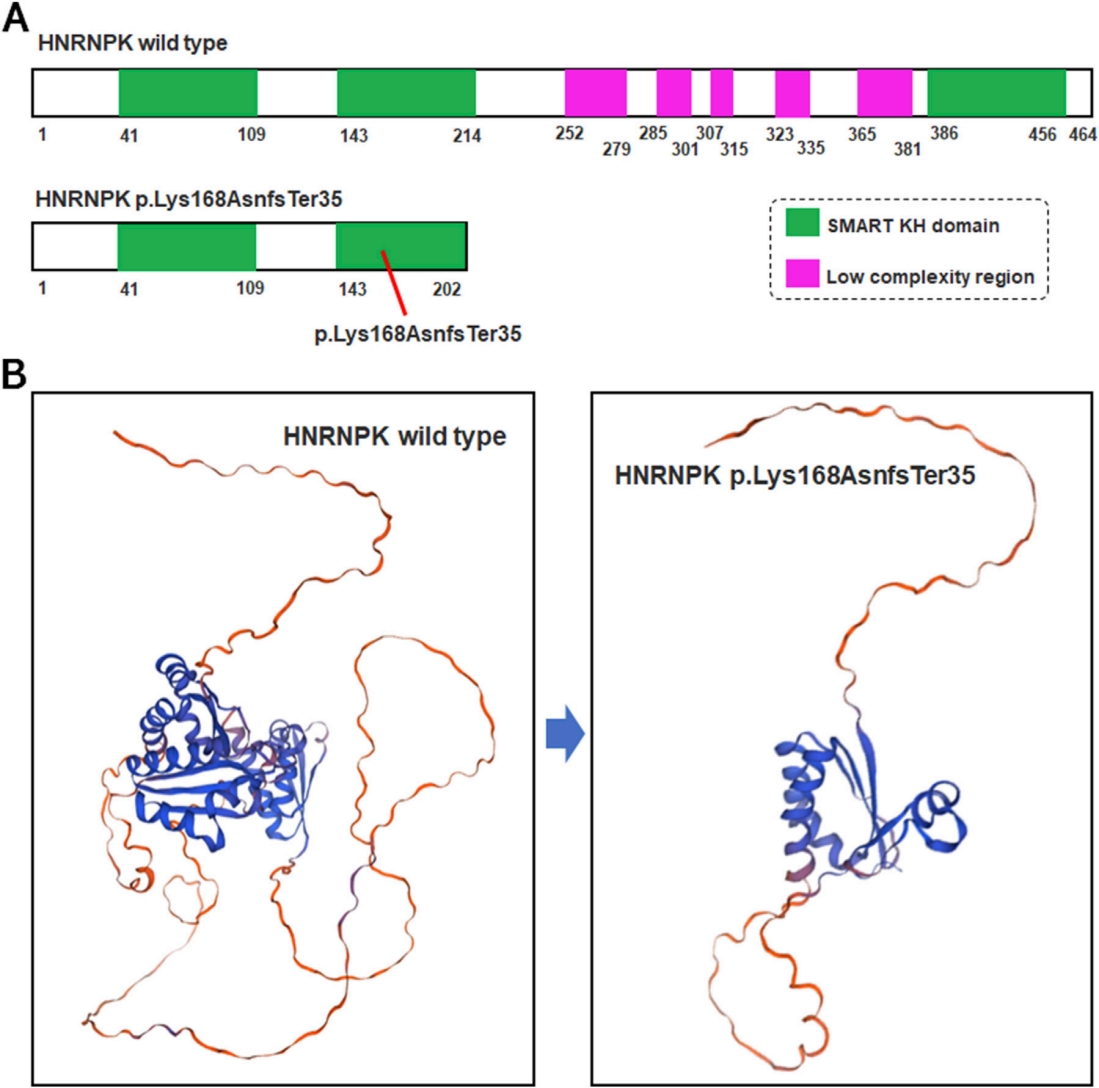
Structural comparison of wild-type and mutant HNRNPK protein. **(A)** Schematic of HNRNPK protein structure. Upper panel: Wild-type HNRNPK (464 aa) with intact SMART KH domains (green) and C-terminal low-complexity regions (purple). Lower panel: Hypothetical p.Lys168AsnfsTer35 mutant (exon nine of 17). **(B)** Predicted 3D structures of HNRNPK. Left panel: Wild-type with canonical fold of functional regions. Right panel: Hypothetical mutant structure-truncation at aa 202 is predicted to disrupt the SMART KH domain and lose the low-complexity region, based on the assumption of mRNA escaping NMD.

The p.Lys168AsnfsTer35 variant in the *HNRNPK* gene is a frameshift variant localized to exon nine of the 17 exons that compose the gene (Figure 3A). This frameshift event introduces a PTC at the genomic level, which activates the cellular frameshift mRNA decay pathway-a highly conserved surveillance mechanism dedicated to eliminating transcripts carrying PTCs (Figure 3B). Functional predictions based on NMD pathway characteristics indicate that approximately 99% of the mutant *HNRNPK* mRNAs harboring this PTC will be recognized and degraded by the NMD machinery. This efficient degradation process is expected to substantially reduce the pool of stable *HNRNPK* mRNA available for ribosome-mediated translation, thereby minimizing the potential for *de novo* protein synthesis from the mutant transcript.

### ClinVar-reported P/LP SNV variants in the *HNRNPK* gene

**FIGURE 4:**
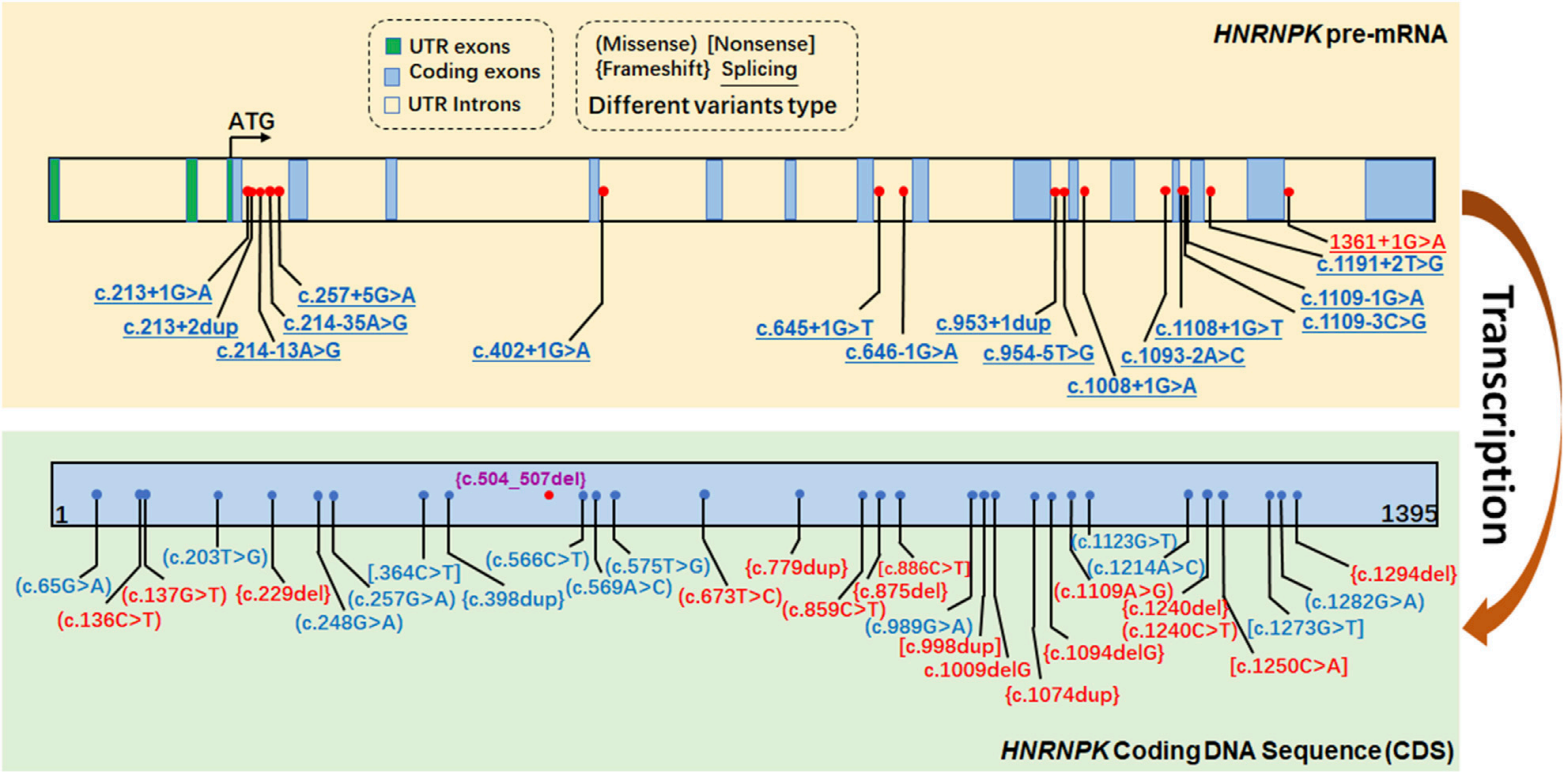
ClinVar-reported P/LP SNV variants in the *HNRNPK* gene. The figure displays variants in the *HNRNPK* gene documented in the ClinVar database, color-coded based on their classification: red for Pathogenic (P), and blue for Likely Pathogenic (LP). Variants highlighted in purple represent those identified and reported in the current study.

To contextualize the variants identified in our study, we analyzed the *HNRNPK* gene variants documented in ClinVar (Figure 4). Variants highlighted in purple represent the variant identified and reported in the current study, c.504-507del. The ClinVar-reported variants span multiple regions of the *HNRNPK* gene. Notably, about 15 intronic P/LP SNP variants in *HNRNPK* lie within ±5 bp of exon boundaries, a pattern consistent with other LoF-associated genes. These intronic variants can impact the splicing of *HNRNPK* pre-mRNA. As proper pre-mRNA splicing is crucial for generating mature mRNA with accurate coding information, disruptions here may interfere with the normal process of translating the *HNRNPK* coding DNA sequence (CDS) into a functional protein. The c.504-507del variant identified in our study is a pathogenic variant. It resides within the *HNRNPK* CDS, which serves as the core template for protein synthesis. Alterations in this region can directly affect the amino acid sequence of the HNRNPK protein, thereby impairing its normal function.

## Discussion

Increased NT and NF are well-established but non-specific soft markers, commonly linked to aneuploidies like Down syndrome (Methods In Medicine CAM, 2023) and Turner syndrome (Gravholt et al., 2017; Yana et al., 2024), but also frequently reported in numerous monogenic disorders such as Noonan syndrome (Tangshewinsirikul et al., 2024), Kabuki syndrome (Sun et al., 2023), and CHARGE syndrome (Hsu et al., 2014). The primary value of this case lies in demonstrating WES as a critical tool for fetuses with nonspecific soft markers, when karyotype and CMA are normal, rather than its specificity for AUKS. This study identified a *de novo* heterozygous frameshift variant in *HNRNPK* (c.504_507del; p.Lys168AsnfsTer35) in a fetus with increased NT (3.4 mm) and NF (9 mm), representing a prenatal diagnosis of AUKS.

This study identified a *de novo* heterozygous frameshift variant in *HNRNPK* (c.504_507del; p.Lys168AsnfsTer35) in an 18 + 1 week fetus with increased NT (3.4 mm) and NF (9 mm), contributing to the prenatal phenotypic spectrum of AUKS. Prior reports have documented *HNRNPK* variants in prenatal cases. A study reported a *HNRNPK* missense variant in a 20-week fetus with NT thickening (3.2 mm) and mild cardiac hypoplasia (Zhang et al., 2024). A slightly more severe prenatal presentation than our case, which lacked cardiac anomalies, aligning with the trend that missense variants may present with earlier structural anomalies compared to frameshift variants in prenatal stages. While Workalemahu *et al.* identified a *HNRNPK* frameshift variant in a 19-week fetus with NF thickening (8 mm) but no major structural anomalies, consistent with our case’s nonspecific soft markers, though those diagnoses were finalized postnatally (Workalemahu et al., 2023). Another study reported six new cases of AUKS, with all patients harboring either *de novo HNRNPK* variants or *de novo* 9q21.32 deletions that include the *HNRNPK* gene (Au et al., 2018). This finding expands the prenatal phenotypic spectrum of AUKS, a neurodevelopmental disorder previously characterized primarily by postnatal features, and underscores the diagnostic value of NT and NF thickening as potential indicators of monogenic disorders like AUKS, particularly in cases with normal karyotype results.

The presence of significant NT and NF thickening in this case, in the absence of major structural anomalies typically screened for prenatally, highlights a crucial diagnostic insight. While NT ≥ 3.0 mm substantially elevates the risk for common aneuploidies (Aygun, 2018), and NF ≥ 6 mm in the second trimester is a strong marker for Down syndrome (Abou Tayoun et al., 2018), this case demonstrates that these soft markers can also herald serious monogenic conditions when aneuploidy is excluded. Our findings suggested that dysfunction of HNRNPK may underlie the abnormal nuchal fluid accumulation observed. HNRNPK is a key regulator of genes involved in diverse processes, including extracellular matrix (ECM) composition and potentially lymphatic development-pathways implicated in nuchal edema formation in other syndromes like Noonan syndrome (Li W. B. et al., 2015). This proposed mechanistic link-where HNRNPK dysfunction disrupts developmental pathways via LoF, which is further supported by the ClinGen curation for *HNRNPK* (https://search.clinicalgenome.org/kb/genes/HGNC:5044), which confirms that LoF and haploinsufficiency are the core drivers of AUKS. Notably, this curation also contextualizes why even a single *de novo* frameshift variant is sufficient to cause AUKS: haploinsufficiency of *HNRNPK* disrupts the delicate balance of RNA processing and gene regulation required for normal fetal development (Liu et al., 2022; Shi et al., 2023). This plausible biological basis for the ultrasound findings in this AUKS case strengthens the argument for considering *HNRNPK* variants in fetuses with isolated NT/NF thickening and normal karyotype/CMA.

Several limitations warrant consideration. Limitations include the single-case design, lack of postnatal neurodevelopmental data, and potential underreporting of mild AUKS cases. As a single case report, the generalizability of NT/NF thickening as specific prenatal markers for AUKS requires validation in larger cohorts. Postnatal follow-up data on neurodevelopmental outcomes for this case were unavailable, limiting our understanding of the full phenotypic correlation. Future studies should aim to define the sensitivity and specificity of these ultrasound markers for AUKS within cohorts of fetuses with isolated NT/NF thickening and negative initial testing. Furthermore, investigating the potential of *HNRNPK*-related epigenetic dysregulation as non-invasive prenatal biomarkers represents a promising avenue for future research. While WES proved diagnostic here, its limitations in prenatal settings include the potential for VUS, challenges in interpreting non-coding variants, incomplete detection of certain variant types, and the ethical complexities surrounding incidental findings and rapid turnaround times. Careful pre- and post-test counseling remains paramount.

The prenatal presentation observed here aligns partially with, yet also contrasts, the established postnatal AUKS phenotype. Large cohort studies report cardiac defects in 50%–60% of postnatal AUKS cases, including atrial septal defects (ASD), ventricular septal defects (VSD), and coarctation of the aorta (Au et al., 2018; Au et al., 1993). However, the fetus in this study exhibited only an isolated hyperechogenic intracardiac focus (EIF), a common finding often considered a benign variant but also associated with aneuploidy and some genetic syndromes. EIF in the left ventricle was confirmed via two orthogonal views; nuchal measurements were repeated twice by a fetal medicine specialist to ensure accuracy. This difference underscores the significant phenotypic variability and potential age-dependent expressivity of AUKS. The absence of major structural cardiac defects *in utero*, despite a severe LoF variant, highlights the incomplete penetrance or variable expressivity of specific features. Factors such as the precise timing of developmental disruption, genetic modifiers, or technical challenges in prenatal cardiac imaging might contribute to this observed heterogeneity (Kingdom and Wright, 2022).

Our case is distinctive for prenatal diagnosis via WES at 18+3 weeks, contrasting with prior reports where diagnosis occurred postnatally despite prenatal anomalies. The *de novo* nature of the variant, consistent with >95% of reported *HNRNPK* pathogenic variants, implies a low recurrence risk for future pregnancies (<1%), though the possibility of undetected parental germline mosaicism necessitates cautious counseling. Prenatal diagnosis enables critical anticipatory guidance and planning for potential postnatal complications commonly seen in AUKS, such as neurodevelopmental delay, hypotonia, feeding difficulties requiring gastrostomy, and craniofacial anomalies like cleft palate. Notably, most intronic pathogenic variants in *HNRNPK* lie within ±5 bp of exon boundaries, affecting splicing, a pattern consistent with other genes associated with loss-of-function. This clarifies that intronic variants in *HNRNPK* are not uncommon but follow a typical pattern of impacting splicing at exon-intron junctions.

Several limitations warrant consideration. As a single case report, the generalizability of NT/NF thickening as specific prenatal markers for AUKS requires validation in larger cohorts. Postnatal follow-up data on neurodevelopmental outcomes for this case were unavailable, limiting our understanding of the full phenotypic correlation. Future studies should aim to define the sensitivity and specificity of these ultrasound markers for AUKS within cohorts of fetuses with isolated NT/NF thickening and negative initial testing. Furthermore, investigating the potential of *HNRNPK*-related epigenetic dysregulation as non-invasive prenatal biomarkers represents a promising avenue for future research (Choufani et al., 2022). While WES proved diagnostic here, its limitations in prenatal settings include the potential for VUS, challenges in interpreting non-coding variants, incomplete detection of certain variant types and the ethical complexities surrounding incidental findings and rapid turnaround times. Careful pre- and post-test counseling remains paramount.

The phenotypic presentation of our prenatal case expands the clinical spectrum of AUKS, which traditionally manifests postnatally with craniofacial anomalies and cardiac defects (Nishtala et al., 2018). In contrast, our fetus exhibited only isolated nuchal thickening and EIF without major structural anomalies, highlighting that AUKS may present with soft markers alone in early gestation. This builds on prior reports of *HNRNPK* variants, which primarily focused on postnatal diagnoses (Miyake et al., 2017), by linking prenatal NT/NF thickening to AUKS. However, as a single case, we acknowledge limitations in establishing NT/NF as specific markers for AUKS; future studies should enroll cohorts with isolated nuchal thickening and negative CMA to determine the prevalence of *HNRNPK* variants. The parents reported anxiety regarding the prior fetal demise and expressed appreciation for the timely WES diagnosis, which informed their decision-making. They consented to publication to aid future prenatal counseling.

In conclusion, this report describes a prenatal diagnosis of AUKS, linking a pathogenic *de novo HNRNPK* frameshift variant to the isolated prenatal findings of increased nuchal translucency and nuchal fold thickening. This expands the recognized prenatal phenotype of AUKS and underscores the critical importance of considering serious monogenic disorders like AUKS in the differential diagnosis of significant soft markers, even in the absence of major structural anomalies or aneuploidy, with WES playing a central role in achieving such diagnoses.

## Data Availability

The datasets presented in this study can be found in online repositories. The names of the repository/repositories and accession number(s) can be found in the article/Supplementary Material.
