# Supplementary material for: Prenatal diagnosis of a de novo pathogenic HNRNPK variant in a Chinese fetus with abnormal ultrasound soft markers: a case report
Source: Front Genet. 2025 Oct 27;16:1661743. doi: 10.3389/fgene.2025.1661743 (PMC12597093; doi:10.3389/fgene.2025.1661743)
Supplement: Supplementary file 1 [file Table1.docx]

**Table S1: Timeline of Key Clinical Events in the Prenatal Care Episode**

| **Gestational Age/Date** | **Key Clinical Event** | **Details** |
| --- | --- | --- |
| October 25, 2024 | Last Menstrual Period (LMP) | Baseline for gestational age calculation |
| 12 weeks | First-trimester screening | Increased nuchal translucency (NT) detected, measuring 3.4 mm (≥95th percentile) |
| 18+1 weeks | Detailed fetal ultrasound + maternal physical examination (PE) + amniocentesis | Ultrasound: Thickened nuchal fold (NF, 9 mm, ≥99th percentile), left ventricular hyperechoic intracardiac focus (EIF, 2 mm, apex-located), normal fetal anatomy, balanced cardiac structure, normal amniotic fluid volume (AFI 192 mm) and umbilical artery Doppler indices  Maternal PE: Stable vital signs (blood pressure 118/72 mmHg, heart rate 76 bpm), uterine size consistent with gestational age, fetal heart tones audible (129 bpm)  Amniocentesis: 18 mL of clear amniotic fluid collected under ultrasound guidance, no blood contamination |
| 18+3 weeks | Sample processing | Genomic DNA extracted from amniotic fluid; peripheral blood samples (5 mL each) collected from both parents for trio genomic analysis |
| 19 weeks | G-banded karyotype result | Normal 46, XY karyotype (resolution: 400–550 bands), ruling out common chromosomal aneuploidies and major structural chromosomal abnormalities |
| 20 weeks | Whole-exome sequencing (WES) + Sanger sequencing initiation | WES identifies a de novo heterozygous nonsense variant in HNRNPK (NM_031263.4: c.504_507del)  Sanger sequencing initiated to validate the identified variant |
| 20+2 weeks | Variant validation + genetic counseling | Sanger sequencing confirms the HNRNPK c.504_507del variant is de novo (absent in parental genomes)  Genetic counseling provided to parents: AUKS diagnosis explained, postnatal prognosis (severe neurodevelopmental delay risk) and recurrence risk (<1%) discussed |
| 21 weeks | Pregnancy termination+post-induction fetal examination | Termination performed via misoprostol (oral 400 μg followed by vaginal 200 μg every 4 hours) under ultrasound guidance  Post-induction gross examination: Fetal craniofacial features consistent with AUKS (broad nasal bridge, micrognathia), no major structural anomalies |
| 21+1 weeks | Post-termination maternal follow-up | Maternal vital signs stable, no uterine contractions, vaginal bleeding, or infection; recovery uneventful |
| 25 weeks (4-week post-termination) | Parental follow-up | Parents report reduced anxiety (self-reported “relief at clarity on genetic cause”) and no depressive symptoms; satisfied with diagnostic timeline and counseling |
