# Supplementary material for: Prenatal diagnosis of a de novo pathogenic HNRNPK variant in a Chinese fetus with abnormal ultrasound soft markers: a case report
Source: Front Genet. 2025 Oct 27;16:1661743. doi: 10.3389/fgene.2025.1661743 (PMC12597093; doi:10.3389/fgene.2025.1661743)
Supplement: Supplementary file 2 [file DataSheet1.pdf]

Topic	Item	Checklist Item Description	Reported on Line
**Title**	**1**	**The diagnosis or intervention of primary focus followed by the words "case report"**	Prenatal diagnosis of a de novo pathogenic HNRNPK variant in a Chinese fetus with abnormal ultrasound soft markers: A case report
**Key Words**	**2**	**2 to 5 key words that identify diagnoses or interventions in this case report (including "case report")**	Nuchal translucency; Prenatal diagnosis; Whole-exome sequencing; De novo mutation; HNRNPK
**Abstract (no references)**	**3a**	**Introduction: What is unique about this case and what does it add to the scientific literature?**	This study reports the prenatal diagnosis of AUKS linked to a de novo HNRNPK nonsense variant (c.504_507del), expanding the prenatal phenotypic spectrum of AUKS (isolated NT/NF thickening + EIF) and underscoring WES's utility in clarifying nonspecific ultrasound findings when standard testing (karyotype) is normal.
	**3b**	**Main symptoms and/or important clinical findings**	A fetus exhibited increased nuchal translucency (NT, 3.4 mm, ≥95th percentile), nuchal fold (NF, 9 mm, ≥99th percentile), and a left ventricular hyperechoic intracardiac focus (EIF, 2 mm, apex-located); no major structural anomalies or growth restriction.
	**3c**	**The main diagnoses, therapeutic interventions, and outcomes**	Diagnosis: Au-Kline syndrome (AUKS) due to de novo HNRNPK nonsense variant (NM_031263.4: c.504_507del; p.Lys168AsnfsTer35). Therapeutic intervention: Pregnancy termination at 21 weeks via misoprostol (oral 400 μg + vaginal 200 μg q4h) under ultrasound guidance. Outcomes: Post-induction exam confirmed AUKS-related craniofacial features (broad nasal bridge, micrognathia); Sanger sequencing validated the de novo variant; structural modeling confirmed truncated protein dysfunction.
	**3d**	**Conclusion—What is the main "take-away" lesson(s) from this case?**	Integrating genomic (WES) and phenotypic (ultrasound) data improves prenatal diagnosis of rare genetic syndromes like AUKS; NT/NF thickening can indicate monogenic disorders even without major structural anomalies,

			supporting WES as a key tool for such cases.
**Introduction**	**4**	**One or two paragraphs summarizing why this case is unique (may include references)**	This case is unique for its prenatal identification of AUKS via a de novo HNRNPK nonsense variant, presenting with isolated NT/NF thickening + EIF (no major structural defects)—expanding AUKS's prenatal phenotype. It addresses a diagnostic gap: NT/NF thickening is often linked to aneuploidies, but this case highlights their role in signaling monogenic disorders (Au et al. 2018; Workalemahu et al. 2023), contributing to understanding HNRNPK-related prenatal manifestations.
**Patient Information**	**5a**	**De-identified patient specific information**	A 30-year-old Han Chinese primigravida (G1P0) with regular 30-day menstrual cycles (LMP: October 25, 2024), BMI 22 kg/m² (normal), no chronic diseases (diabetes/hypertension), no teratogen exposure (smoking/alcohol/medications/radiation) during pregnancy.
	**5b**	**Primary concerns and symptoms of the patient**	The patient's primary concerns: Prior fetal demise (16 weeks' gestation, undetermined cause) and new prenatal ultrasound findings (increased NT/NF + EIF), prompting referral to a fetal medicine clinic for further evaluation.
	**5c**	**Medical, family, and psycho-social history including relevant genetic information**	Medical history: Mother had one prior pregnancy (1 year prior) with fetal demise at 16 weeks (no structural anomalies on post-mortem, no genetic testing). Family history: No known genetic disorders, consanguinity, or pregnancy losses in either parent's immediate family; both parents (30 years old) have normal developmental histories and no craniofacial/cardiac anomalies. Psycho-social history: Parents reported high anxiety due to prior demise, seeking clarity on fetal genetic health for reproductive decision-making.
	**5d**	**Relevant past interventions with outcomes**	Prior pregnancy (1 year prior): First-trimester NT screening (normal, 1.8 mm); declined invasive testing; fetal demise at 16 weeks (post-mortem: no structural anomalies, no genetic testing). Current pregnancy: Declined serum quadruple screening (low confidence in rare syndrome detection); opted for

			amniocentesis + WES after genetic counseling.
**Clinical Findings**	**6**	**Describe significant physical examination (PE) and important clinical findings.**	Maternal PE (18+1 weeks): Vital signs stable (BP 118/72 mmHg, HR 76 bpm); uterine size consistent with gestational age; no uterine tenderness/abnormal vaginal discharge; fetal heart tones audible via Doppler (129 bpm).<br>Fetal Findings: Biometry (18+1 weeks): Biparietal diameter 47 mm, head circumference 188 mm, abdominal circumference 172 mm, femur length 34 mm, humerus length 32 mm, transverse cerebellar diameter 22 mm, lateral ventricular width 7 mm (all normal for gestation). Structural evaluation: Normal skull, spine, lips, extremities, stomach, kidneys, bladder, umbilical cord insertion. Cardiac: Balanced four-chamber view, aortic diameter 2.2 mm, pulmonary artery diameter 2.3 mm (ratio 1.0), regular rhythm (129 bpm), left ventricular EIF (2 mm, apex-located). Placenta: Anterior, 21 mm thick, Grade I maturity. Amniotic fluid: AFI 192 mm (normal: Right Upper Quadrant 61 mm, Left Upper Quadrant 36 mm, Right Lower Quadrant 57 mm, Left Lower Quadrant 38 mm). Umbilical artery Doppler: Pulsatility Index 1.29, Resistance Index 0.71, Systolic/Diastolic ratio 3.47 (all normal).
**Timeline**	**7**	**Historical and current information from this episode of care organized as a timeline**	(Table S1: Timeline of Key Clinical Events)<br>-October 25, 2024: Last menstrual period (LMP)<br>-12 weeks' gestation: First-trimester screening, increased NT (3.4 mm) detected<br>-18+1 weeks' gestation: Detailed fetal ultrasound (NF 9 mm + left ventricular EIF+normal anatomy); maternal PE (stable vital signs); amniocentesis performed (18 mL clear fluid, no contamination)<br>-18+3 weeks' gestation: Amniotic fluid DNA extraction; parental peripheral blood samples collected<br>-19 weeks' gestation: G-banded karyotype result (46, XY, normal)

			-20 weeks' gestation: WES identifies HNRNPK c.504_507del variant; Sanger sequencing initiated for validation<br>-20+2 weeks' gestation: Sanger sequencing confirms de novo variant; genetic counseling provided to parents<br>-21 weeks' gestation: Pregnancy termination via misoprostol; post-induction fetal gross examination<br>-21+1 weeks' gestation: Post-termination maternal follow-up (no complications)<br>-25 weeks' gestation (4-week post-termination): Parental follow-up (reduced anxiety, no depression)
**Diagnostic Assessment**	**8a**	**Diagnostic testing (such as PE, laboratory testing, imaging, surveys).**	1. Prenatal ultrasound (12 weeks: NT measurement; 18+1 weeks: fetal anatomy, NF, EIF, biometry, placenta, amniotic fluid, Doppler).<br>2. Maternal PE (18+1 weeks: vital signs, uterine size, fetal heart tones).<br>3. Genetic testing: G-banded karyotype (amniotic fluid, 400–550 band resolution, normal 46, XY); whole-exome sequencing (WES: Agilent SureSelect Human All Exon V6 capture, BGI-seq2000 platform, average depth 263×, 98.75% target coverage >20×); Sanger sequencing (variant validation, ABI 3730xl analyzer).<br>4. Bioinformatic analysis: Phyre2 (protein structural modeling), ANNOVAR (variant annotation), GATK (variant calling), ACMG/AMP guidelines (variant pathogenicity classification).
	**8b**	**Diagnostic challenges (such as access to testing, financial, or cultural)**	1. Nonspecific ultrasound findings: NT/NF thickening + EIF overlap with >20 monogenic syndromes (e.g., Noonan, CHARGE) and aneuploidies, complicating initial suspicion (Liu et al. 2022).<br>2. Rarity of prenatal AUKS: Few reported prenatal cases (Workalemahu et al. 2023; Zhang et al. 2024), limiting prior clinical reference.<br>3. Testing sequence delay: Need to rule out aneuploidies via karyotype first,

			delaying WES initiation until normal karyotype confirmation.<br>4. Variant interpretation: Lack of functional assays (e.g., RNA sequencing) to confirm nonsense-mediated mRNA decay (NMD) activation, relying on in silico predictions (Miyake et al. 2017).
	**8c**	**Diagnosis (including other diagnoses considered)**	Final Diagnosis: Au-Kline syndrome (AUKS) due to de novo heterozygous nonsense variant in HNRNPK (NM_031263.4: c.504_507del; p.Lys168AsnfsTer35), classified as pathogenic per ACMG/AMP guidelines.<br>Differential Diagnoses Ruled Out:<br>1. Aneuploidies (Down syndrome, Turner syndrome): Ruled out via normal 46, XY karyotype.<br>2. Noonan syndrome: Ruled out by absence of cardiac defects (e.g., pulmonary stenosis) and negative WES for PTPN11/RAS pathway variants.<br>3. Kabuki syndrome: Ruled out by absence of typical craniofacial features (e.g., long palpebral fissures) and negative WES for KMT2D/KDM6A variants.<br>4. CHARGE syndrome: Ruled out by normal fetal anatomy (no coloboma, choanal atresia) and negative WES for CHD7 variants.
	**8d**	**Prognosis (such as staging in oncology) where applicable**	Prognosis is guarded: HNRNPK nonsense variants (like c.504_507del) are severe loss-of-function (LoF) variants, associated with 80% risk of severe postnatal neurodevelopmental delay, 50% risk of feeding difficulties requiring gastrostomy, and 40% risk of craniofacial anomalies (Au et al. 2018). Prenatal soft markers (NT/NF thickening) correlate with postnatal neurodevelopmental risk rather than acute prenatal structural severity, supporting the clinical value of early genetic diagnosis for anticipatory care.
**Therapeutic Intervention**	**9a**	**Types of therapeutic intervention (such as pharmacologic, surgical, preventive, self-care)**	Primary intervention: Medical termination of pregnancy (therapeutic abortion) for confirmed AUKS with severe postnatal prognosis, per parental informed decision.

	**9b**	**Administration of therapeutic intervention (such as dosage, strength, duration)**	Intervention administered at 21 weeks' gestation, following institutional second-trimester termination protocol: Oral misoprostol 400 μg (initial dose), followed by vaginal misoprostol 200 μg every 4 hours until uterine contractions initiated. Procedure performed under real-time ultrasound guidance to monitor fetal status and avoid uterine injury. Post-procedural monitoring (30 minutes) for uterine contractions, vaginal bleeding, or maternal hypotension.
	**9c**	**Changes in therapeutic intervention (with rationale)**	No changes in therapeutic intervention: The diagnostic pathway (karyotype → WES → Sanger validation) was completed without delays (2 weeks from amniocentesis to variant confirmation), and parental decision to terminate remained consistent after genetic counseling (informed of recurrence risk <1% and postnatal care burdens).
**Follow-up and Outcomes**	**10a**	**Clinician and patient-assessed outcomes (if available)**	Clinician-Assessed Outcomes:<br>-Post-termination fetal gross examination: Confirmed AUKS-related craniofacial features (broad nasal bridge, micrognathia) (Figure 2C/D); no major structural anomalies (consistent with prenatal ultrasound).<br>-Maternal post-procedural (21+1 weeks): No uterine contractions, vaginal bleeding, or infection; vital signs stable.<br>Patient-Assessed Outcomes (4-week post-termination):<br>-Parents reported reduced anxiety (patient-quoted "relief at clarity on genetic cause") and no depressive symptoms.<br>-Parents expressed satisfaction with the diagnostic timeline (2 weeks from amniocentesis to result) and counseling quality.
	**10b**	**Important follow-up diagnostic and other test results**	-No additional diagnostic tests needed: Sanger sequencing had confirmed the de novo HNRNPK variant; karyotype/CMA ruled out aneuploidies/copy-number variants.<br>-Parental carrier screening: Offered for HNRNPK and other

			neurodevelopmental disorder (NDD) genes (e.g., SCN1A, CFTR) but declined by parents (low recurrence risk perception).<br>-Maternal menstrual cycle: Resumed by 6 weeks post-termination (normal flow, no dysmenorrhea).
	**10c**	**Intervention adherence and tolerability (How was this assessed?)**	Adherence: The patient fully adhered to the termination protocol (completed all misoprostol doses) and follow-up appointments (post-procedural check, 4-week follow-up). Assessed via clinical documentation (dose administration records, appointment attendance).<br>Tolerability: Good tolerability; mild uterine cramping (managed with oral acetaminophen 500 mg q6h for 24 hours) and minimal vaginal spotting (resolved within 72 hours). No severe adverse effects (heavy bleeding, fever, infection) reported. Assessed via maternal self-report and clinical exam (vital signs, uterine tenderness, vaginal discharge) at follow-up.
	**10d**	**Adverse and unanticipated events**	No adverse or unanticipated events occurred during the diagnostic process (amniocentesis, WES, Sanger sequencing) or therapeutic intervention (termination):<br>-Amniocentesis: No blood contamination, uterine contractions, or vaginal bleeding post-procedure.<br>-Termination: No uterine rupture, infection, or maternal hypotension.<br>-Post-termination: No delayed complications (e.g., endometritis, abnormal uterine bleeding).
**Discussion**	**11a**	**A scientific discussion of the strengths AND limitations associated with this case report.**	Strengths:<br>1. Trio WES (fetus + parents) confirmed de novo variant origin, reducing false-positive risk (a common prenatal genomics pitfall) (Liu et al. 2022).<br>2. Multi-modal validation: Sanger sequencing (variant), ultrasound (phenotype), post-induction exam (craniofacial features) correlated genotype with

			phenotype, critical for AUKS diagnosis (nonspecific prenatal features) (Choufani et al. 2022).<br>3. Detailed clinical documentation: Comprehensive maternal PE, fetal ultrasound, and follow-up data align with CARE guidelines, enhancing reproducibility.<br>Limitations:<br>1. Lack of functional assays: No RNA sequencing/protein expression analysis to confirm NMD activation; reliance on in silico predictions (Phyre2) weakens mechanistic claims (Miyake et al. 2017).<br>2. No postnatal follow-up: Pregnancy termination precluded assessment of whether mild prenatal phenotype would progress to severe postnatal AUKS (e.g., neurodevelopmental delay).<br>3. Selection bias: Cases with isolated soft markers + normal karyotype are less likely to undergo WES in clinical practice, limiting generalizability (Shi et al. 2023).<br>4. Single-case design: Cannot establish NT/NF thickening as "specific" AUKS markers; requires larger cohorts for validation.
	**11b**	**Discussion of the relevant medical literature with references.**	AUKS, caused by heterozygous HNRNPK LoF variants, was first described in 2015 (Au et al. 2015) with postnatal features (developmental delay, craniofacial anomalies). Prenatal AUKS cases remain rare: Workalemahu et al. (2023) reported a 19-week fetus with HNRNPK frameshift variant and isolated NF thickening (8 mm), similar to our case's nonspecific soft markers; Zhang et al. (2024) described a 20-week fetus with HNRNPK missense variant and NT thickening + cardiac hypoplasia (slightly more severe prenatal presentation), aligning with our finding that missense variants (milder LoF) may present with earlier structural anomalies than nonsense variants (severe LoF) (Gillentine et

			al. 2021). NT ≥3.0 mm increases monogenic disorder risk 2–3-fold (Aygun 2018), and NF ≥6 mm is linked to rare syndromes (Abou Tayoun et al. 2018)—our case supports these markers as triggers for WES. WES yields 8–19% incremental diagnosis in fetuses with soft markers (Liu et al. 2022; Shi et al. 2023), underscoring its utility here.
	**11c**	**The scientific rationale for any conclusions (including assessment of possible causes)**	Conclusions are rationalized by the de novo variant's pathogenicity (truncated protein, disrupted KH domain), its absence in population databases, and alignment with AUKS pathogenesis. Possible causes include spontaneous mutation leading to the HNRNPK variant.
	**11d**	**The primary "take-away" lessons of this case report (without references) in a one paragraph conclusion**	NT and NF thickening can indicate rare monogenic disorders like AUKS even without major structural anomalies. Integrating WES with prenatal imaging improves diagnosis of such syndromes, highlighting the need for genetic testing in fetuses with abnormal soft markers.
Patient Perspective	**12**	**The patient should share their perspective in one to two paragraphs on the treatment(s) they received.**	NA
Informed Consent	**13**	**Did the patient give informed consent? Please provide if requested**	Yes. Written informed consent was obtained from both parents prior to sample collection.
